# Genome sequence of a bovine respiratory syncytial virus strain from a cow in Tennessee

**DOI:** 10.1128/mra.00072-25

**Published:** 2025-04-28

**Authors:** Ola K. Elsakhawy, Ashkan Roozitalab, Mohamed A. Abouelkhair

**Affiliations:** 1 Diagnostic Medicine and Pathobiology, Shreiber School of Veterinary Medicine, Rowan University 3536, Glassboro, New Jersey, USA; 2 Department of Biomedical and Diagnostic Sciences, University of Tennessee 204922, Knoxville, Tennessee, USA; DOE Joint Genome Institute, Berkeley, California, USA

**Keywords:** Tennessee, BRSV, respiratory viruses, metagenomics, veterinary diagnostics

## Abstract

We report the genome sequence of a bovine respiratory syncytial virus strain (hereafter referred to as BRSV_23), identified in a cow in Tennessee. This genome sequence updates the currently circulating BRSV field strains in the United States, providing insights into viral evolution and epidemiology to improve diagnostics and control strategies.

## ANNOUNCEMENT

Bovine respiratory syncytial virus (BRSV) is an enveloped virus with a single-stranded, negative-sense RNA genome that belongs to the *Orthopneumovirus* genus within the *Pneumoviridae* family (1). BRSV is a major viral pathogen in cattle, especially in young calves, and is a leading cause of respiratory illness (2). BRSV targets epithelial cells of the respiratory tract, inducing syncytia (fusion of infected cells), which leads to tissue damage, inflammation, and mucus buildup. This damage impedes airflow and creates an environment for secondary bacterial infections, further complicating the disease (2). Metagenomic sequencing improves diagnostic performance for respiratory infections by allowing for the unbiased detection of a wide range of pathogens, including bacteria, viruses, and fungi, in a single test (3, 4).

**Fig 1:**
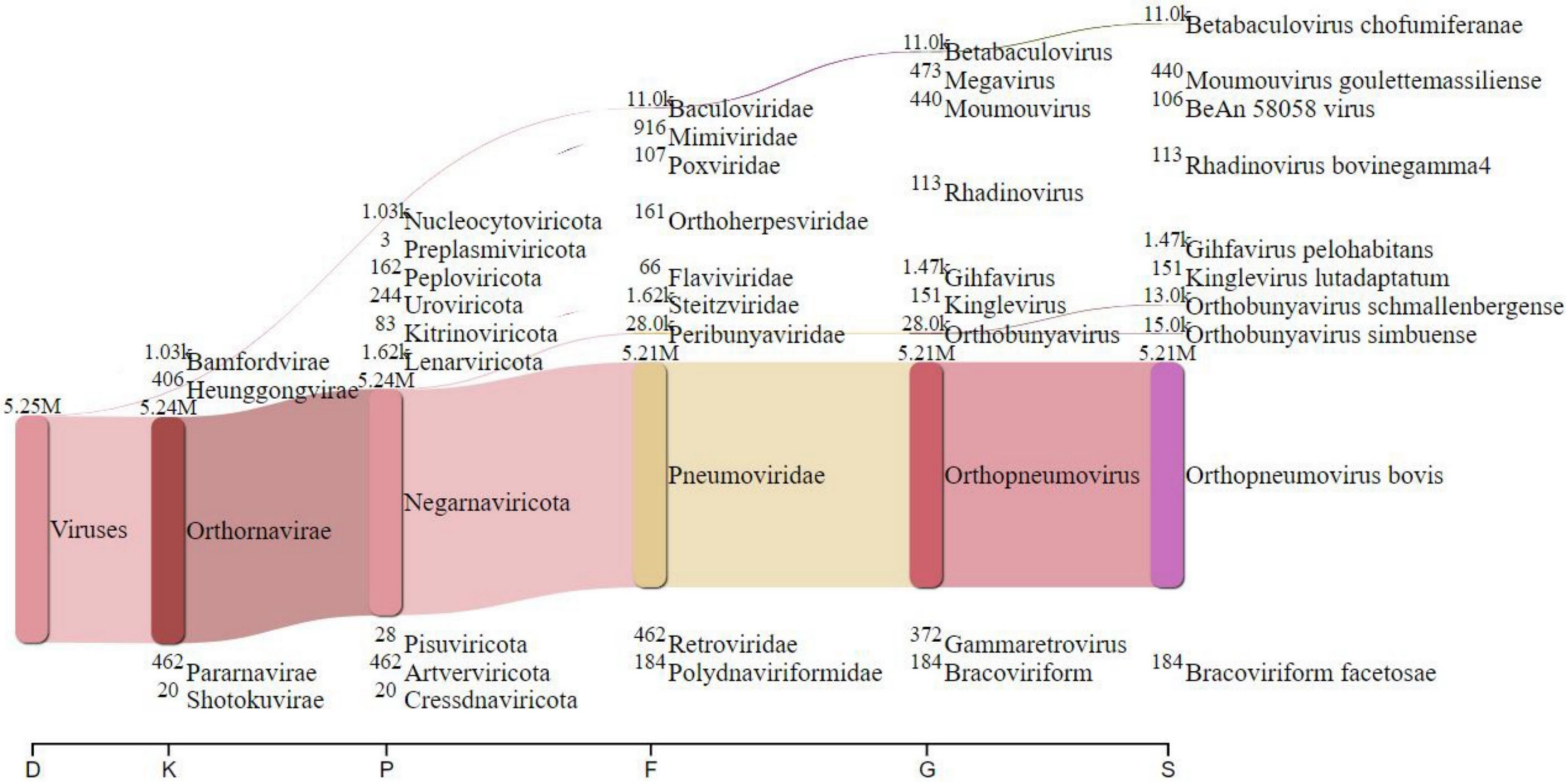
Sankey diagram representing the Kraken2 report. The flow width corresponds to the number of reads, and the number above each node indicates the *K-mer* hits. A rank code was applied to denote domain (D), kingdom (K), phylum (P), family (F), genus (G), or species (S). Out of the total 5,253,956 viral reads analyzed, a substantial proportion of 5,210,973 reads (99.18%) were classified as BRSV.

Lung tissue was obtained from a 4-month-old cow that had been euthanized due to respiratory disease in the summer of 2023 and was immediately analyzed using an in-house respiratory diagnostic quantitative polymerase chain reaction (qPCR) panel on a QuantStudio 3 system at the University of Tennessee College of Veterinary Medicine Diagnostic Laboratory. The panel, which tests for bovine respiratory syncytial virus, bovine viral diarrhea virus (BVDV), and infectious bovine rhinotracheitis (IBR), was positive for BRSV and negative for BVDV and IBR. For sequencing, 1 g of lung tissue was suspended in 0.5 mL of phosphate-buffered saline and thoroughly homogenized using a FastPrep-24 homogenizer (MP Biomedicals, USA). The RNA was extracted and purified using MagMAX Viral/Pathogen Nucleic Acid Isolation Kit (Thermo Fisher Scientific, USA). Samples were DNAse treated with Invitrogen DNAse (RNAse free) at a final concentration of 0.1 U/µL. The RNA quantity and quality were evaluated using a NanoDrop 2000 (Thermo Fisher Scientific, USA) and Qubit flex fluorometer (Fisher, Waltham, MA). Library preparation was performed using Illumina’s Stranded Total RNA Prep Ligation with Ribo-Zero Plus kit and 10 bp unique dual indices. Sequencing was performed using a NovaSeq X Plus platform, generating 150 bp paired-end reads, with a total of 37.8 million reads. Demultiplexing, quality control, and adapter trimming were performed with bcl-convert (v4.2.4). Metagenomics data analysis was performed using the Sunbeam pipeline (version 4.7.0) (5). Taxonomic classification was performed using Kraken2 (version 2.1.3) on Illumina reads (6, 7). The Kraken2 result was subsequently visualized using Pavian (version 1.0) with rank codes representing the taxonomic ranks of domain, kingdom, phylum, family, genus, or species (Fig. 1) (8). Out of the total 5,253,956 viral reads analyzed, a substantial proportion of 5,210,973 reads (99.18%) were classified as bovine respiratory syncytial virus. Then viral reads were assembled into contigs using MEGAHIT (version 1.2.9) (9, 10). The obtained genome of bovine respiratory syncytial virus was 15,146 nucleotides in size (GC content: 33.5%) and was annotated using Viral Annotation Pipeline and iDentification (VAPiD; version 1.2) (11). The final genome read coverage was 99035.2×. BRSV_23 showed identities of 86%–99% to the known bovine respiratory syncytial virus strains from GenBank (Table 1). All the sequence analysis described in this study was conducted utilizing the Jetstream2 cloud computing resource which is supported by the National Science Foundation (12). Default parameters were used except where otherwise noted.

**TABLE 1:** Publicly available genome sequences of BRSV strains

Accession	Strain/isolate	Host	% Identity of BRSV_23 to each strain	Coverage	Length (bp)	Completeness	Country	Collection_ Date
OM860285.1	BRSV/Bovine/Italy/strain 48036/MA/2018	*Bos taurus*	95	100	15,139	Complete	Italy	2018
OP137030.1	BO/SWUN-1/21/CH	*Bos taurus*	98	100	15,150	Complete	China	2021
OP137031.1	BO/SWUN-2/21/CH	*Bos taurus*	98	100	15,145	Complete	China	2021
OP137032.1	BO/SWUN-3/20/CH	*Bos taurus*	98	100	15,142	Complete	China	2020
OP137034.1	BO/SWUN-5/22/CH	*Bos taurus*	98	100	15,145	Complete	China	2022
OP609672.1	YAK/SWUN-1/21/CH	*Bos grunniens*	98	100	15,143	Complete	China	2021
OP715679.1	A_BRSVDKp7	*Bos taurus*	95	96	14,561	Partial	Denmark	2001
OP715686.1	C_7088BALd7	*Bos taurus*	96	96	14,563	Partial	Sweden	2012
OP715687.1	C_7088NSd6	*Bos taurus*	96	96	14,563	Partial	Sweden	2012
OP715692.1	D_3982BALd6	*Bos taurus*	96	96	14,625	Partial	France	2014
OP715694.1	D_3996BALd7	*Bos taurus*	96	96	14,625	Partial	France	2014
OP715706.1	F_8531BALd5	*Bos taurus*	95	95	14,563	Partial	Sweden	2018
OP715707.1	F_8537NSd4	*Bos taurus*	94	60	14,563	Partial	Sweden	2018
OP715709.1	F_SnookBAL2018	*Bos taurus*	96	96	14,563	Partial	United Kingdom	2018
OP715710.1	G_2035BALd7	*Bos taurus*	94	96	14,562	Partial	Sweden	2018
OP715713.1	G_2081BALd7	*Bos taurus*	94	96	14,556	Partial	Sweden	2018
OP715719.1	O_AI12U20	*Bos taurus*	94	96	14,562	Partial	Sweden	2020
OP715722.1	O_AJ22U20	*Bos taurus*	94	96	14,562	Partial	Sweden	2020
OP715724.1	O_BE11J20	*Bos taurus*	94	96	14,562	Partial	Sweden	2020
OP715725.1	O_BK11J20	*Bos taurus*	94	96	14,562	Partial	Sweden	2020
OP020146.1	BRSV_NSWL4	*Bos taurus*	86	87	13,416	Partial	Australia	2019
OM328114.1	BRSV\KS\467\2021	*Bos taurus*	99	100	15,122	Complete	USA	2021
OM328115.1	BRSV\KS\090\2021	*Bos taurus*	98	96	14,801	Partial	USA	2021
MT861050.1	DQ	*Bos taurus*	96	100	15,151	Complete	China	2018
MG947594.1	BRSV_Sweden_HPIG-SLU-620-Lovsta_2016	*Bos taurus*	94	100	15,140	Complete	Sweden	2016
KU159366.1	USII/S1	*Bos taurus*	99	100	15,123	Complete	USA	2015

This genome sequence provides an essential update on the currently circulating BRSV field strains in the United States, shedding light on viral evolution and epidemiology. This knowledge helps to improve diagnostic tools, vaccine design, and management techniques for more successful BRSV control.

## Data Availability

This Whole Genome Shotgun project has been deposited in GenBank under accession no. PQ499118. The version described in this paper is the first version, PQ499118.1 and in the Sequence Read Archive under accession number SRR31015819.
